# Genomes of Three Closely Related Caribbean Amazons Provide Insight for Species History and Conservation

**DOI:** 10.3390/genes10010054

**Published:** 2019-01-16

**Authors:** Sofiia Kolchanova, Sergei Kliver, Aleksei Komissarov, Pavel Dobrinin, Gaik Tamazian, Kirill Grigorev, Walter W. Wolfsberger, Audrey J. Majeske, Jafet Velez-Valentin, Ricardo Valentin de la Rosa, Joanne R. Paul-Murphy, David Sanchez-Migallon Guzman, Michael H. Court, Juan L. Rodriguez-Flores, Juan Carlos Martínez-Cruzado, Taras K. Oleksyk

**Affiliations:** 1 Department of Biology, University of Puerto Rico at Mayaguez, Mayaguez, PR 00680, USA; sofiia.kolchanova@upr.edu (S.K.); kig2007@med.cornell.edu (K.G.); wwolfsberger@oakland.edu (W.W.W.); audrey.majeske@upr.edu (A.J.M.); juancarlos.martinez@upr.edu (J.C.M.-C.); 2 Department of Biology, University of Konstanz, 78464 Konstanz, Germany; 3 Theodosius Dobzhansky Center for Genome Bioinformatics, St. Petersburg State University, 199034 St. Petersburg, Russia; ad3002@gmail.com (A.K.); pdobrynin@gmail.com (P.D.); gaik.tamazian@gmail.com (G.T.); 4 Department of Genetic Medicine, Weill Cornell Medical College, New York, NY 10021, USA; jur2014@med.cornell.edu; 5 Department of Biological Sciences, Oakland University, 118 Library Drive, Rochester, MI 48309, USA; 6 Department of Biological Sciences, Uzhhorod National University, 88000 Uzhhorod, Ukraine; 7 Beaumont BioBank, William Beaumont Hospital, Royal Oak, MI 48073, USA; 8 Conservation Program of the Puerto Rican Parrot, U.S. Fish and Wildlife Service, Rio Grande, PR 00745, USA; jafet_velez@fws.gov; 9 The Recovery Program of the Puerto Rican Parrot at the Rio Abajo State Forest, Departamento de Recursos Naturales y Ambientales de Puerto Rico, Arecibo, PR 00613, USA; el.cotorro.electrico@gmail.com; 10 Department of Medicine and Epidemiology, School of Veterinary Medicine, University of California Davis, Davis, CA 95616, USA; paulmurphy@ucdavis.edu (J.R.P.-M.); guzman@ucdavis.edu (D.S.-M.G.); 11 Program in Individualized Medicine (PrIMe), Pharmacogenomics Laboratory, Department of Veterinary Clinical Sciences, College of Veterinary Medicine, Washington State University, 100 Grimes Way, Pullman, WA 99164, USA; michael.court@wsu.edu

**Keywords:** genomics, parrots, birds, heterozygosity, Puerto Rican parrot, Cuba, Hispaniola, conservation, demography

## Abstract

Islands have been used as model systems for studies of speciation and extinction since Darwin published his observations about finches found on the Galapagos. Amazon parrots inhabiting the Greater Antillean Islands represent a fascinating model of species diversification. Unfortunately, many of these birds are threatened as a result of human activity and some, like the Puerto Rican parrot, are now critically endangered. In this study we used a combination of de novo and reference-assisted assembly methods, integrating it with information obtained from related genomes to perform genome reconstruction of three amazon species. First, we used whole genome sequencing data to generate a new de novo genome assembly for the Puerto Rican parrot (*Amazona vittata*). We then improved the obtained assembly using transcriptome data from *Amazona ventralis and* used the resulting sequences as a reference to assemble the genomes Hispaniolan (*A. ventralis*) and Cuban (*Amazona leucocephala*) parrots. Finally, we, annotated genes and repetitive elements, estimated genome sizes and current levels of heterozygosity, built models of demographic history and provided interpretation of our findings in the context of parrot evolution in the Caribbean.

## 1. Introduction

The Bird 10,000 Genomes (B10K) Project resulted in a large number of genomic sequences that are being quickly assembled and incorporated into studies on evolution, ecology, population genetics, neurobiology, development and conservation [1,2,3]. Genome-wide sequencing and assembly has expanded to the point that it allows for completion of the genome-based phylogeny of all birds. Attention has recently started to shift away from representation of the overall bird phylogeny to instead filling in gaps and resolving specific lineages. A narrowed focus on speciation and adaptation processes on the species level can allow for decoding of the links between genotypes and phenotypes; determining genetic, evolutionary, biogeographical and biodiversity relationships across species; and evaluation of how various ecological factors affect avian evolution [4]. Finally, by focusing on groups that include endangered species, genome studies provide the means to elucidate the conservation issues that would help in our efforts to preserve biodiversity. Neotropical parrots represent a fascinating group that includes many species with endangered conservation status that have not yet been represented in whole-genome phylogenetic analyses [2,3].

Islands became an important source of evolutionary ideas [5,6,7]. Since Darwin’s Voyage on the Beagle, they provided valuable model systems for fundamental studies of migration, diversification and extinction [5,8]. Amazon parrots (*Amazona* sp.) that inhabit the Greater Antillean Islands are a fascinating example of speciation on islands, in many ways similar to that of Darwin’s finches in the Galapagos [9]. Several attempts have been made in the past to shed light on evolution and speciation of these birds based on morphological [10,11] and molecular data [12] but the picture still falls shy of full resolution. Considering the significance of parrots to these islands’ history and ecology [13,14,15,16], we believe it is important to understand how these species came to be and how they adapted to specific island environments. In this article, we focus on the clade of amazon parrots that originated in Central America and spread across the Caribbean islands of Cuba, Hispaniola and Puerto Rico [10,11].

Parrots have long been thought to have first originated in and diversified from, Gondwana, based on current distribution across the southern continents that formerly composed this giant ancient supercontinent [17]. Initial biogeographic analyses, based on multi-loci phylogenies, extensive taxa sampling and different analytical approaches, support a hypothesis of origin and initial diversification in Gondwana during the Cretaceous [18]. Consequently, separation of *Arinae* (New World parrots that include amazons, macaws, conures and parakeets) from other groups of parrots was associated with drift of major Gondwana plates around 35 Mya [18]. Accordingly, *Amazona* parrots were thought to have split from all the other neotropical parrots around 23 Mya. Other, more recent and robustly supported independent phylogenomic analyses [3,19,20], as well as fossil evidence [21,22], support post-Gondwana divergence of stem Psittaciformes from Psittacopasseres between 55–60 Mya. These studies estimate the earliest divergence of crown group Psittaciformes (*Nestor*-Psittacidae) to have occurred between 42–32 Mya and the divergence of New World parrots from others as recently as 14 Mya [3,19,20,21,22,23].

**Figure 1:**
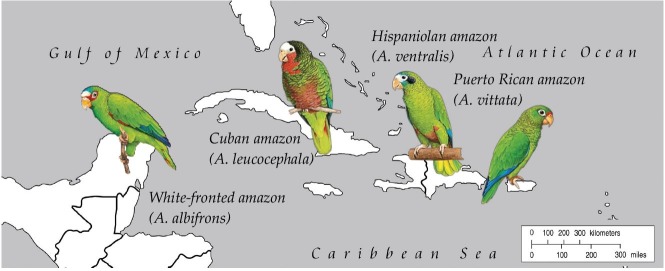
Amazon parrots included in this study (*Amazona leucocephala, A. ventralis* and *A. vittata*) may all have originated from Central America, where the white-fronted amazon (*A. albifrons*) can be found today (modified from Kolchanova (2018) [26]).

The parrots on the Greater Antilles appear closely related to the small *A. albifrons* of Central America (Figure 1) and there may have been two separate dispersal events to these islands, one directly to Jamaica and one to Cuba, followed by the stepping stone dispersal from island to island, as far as to Puerto Rico [10,11,24]. Unfortunately, the sequence and timing of many of these significant evolutionary events are inferred from limited molecular or geological data. With the arrival of genome sequencing and new fossil data, the history of speciation is being refined using the additional evidence. Evolutionary history of the *Amazona* clade was previously assessed using a small set of genetic markers (cytochrome B gene, COI gene etc.) producing the first molecular phylogeny [11,23]. This analysis did not provide speciation times and left other unresolved issues due to the insufficient amount of information: at least two contradicting colonization scenarios for the speciation order among the four major Caribbean islands (Cuba, Hispaniola, Jamaica and Puerto Rico) have been proposed [10,11]. Even if the full mitogenomic sequences were used, an analysis based solely on mtDNA may not have been sufficient due to the incomplete lineage sorting and subsequent gene flow between the islands, which can interfere with proper interpretation of phylogenetic trees [25]. Nuclear genomes or at least multiple nuclear genes, along with mitochondrial data, should be incorporated into the analysis to rule out misinterpretations and to reconstruct events leading to parrot speciation on islands.

So far, the only publicly available genome from the Caribbean amazon clade (Figure 1) has been from the Puerto Rican parrot (*Amazona vittata*): a short-read assembly with only 76% coverage that probably included a number of mis-assemblies [27]. The critically endangered *A. vittata* is the only surviving indigenous parrot species anywhere in the U.S. [27]. Once abundant throughout the island of Puerto Rico, its drastic population decline followed the decimation of the old-growth forest [28]. Despite early DNA fingerprinting efforts [29,30], the genetic consequences of the severe population bottleneck, as well as the population expansion associated with the recent recovery, have not been fully evaluated and a more comprehensive analysis of the genome on the population and species levels is needed. Further detailed research on *A. vittata* conservation genomics is necessary to provide data and better tools to study inbreeding depression, mutation and adaptation to captivity [31,32]. 

In the current study, we used additional genome wide and transcriptome data to improve that assembly, as well as to assemble and annotate the genomic sequences of two additional amazon species from the Caribbean: the Cuban amazon (*A. leucocephala*) and the Hispaniolan amazon (*A. ventralis*). Using both genome and transcriptome data we have generated the improved de novo assembly of the *A. vittata* genome, performed reference-assisted assembly for two other closely related Caribbean amazon species, annotated protein-coding genes and repeats, analyzed demographic history and genomic levels of heterozygosity and discussed our results in the context of conservation biology of these species.

## 2. Materials and Methods

### 2.1. Samples

Blood samples for DNA sequencing from female Puerto Rican (*A. vittata)* and Hispaniolan parrots (*A. ventralis)* were obtained during routine veterinary procedures from birds housed at the US Fish and Wildlife Service “Iguaca” Aviary, a captive-breeding facility for the Puerto Rican parrot near El Yunque National Rainforest in Puerto Rico. All procedures were approved by the University of Puerto Rico at Mayagüez Institutional Animal Care and Use Committee (IACUC#201109.1) and were in accordance with the guidance for the Endangered Species Act. The Cuban parrot (*A. leucocephala*) DNA sample was extracted from the living cell cultures of the Frozen Zoo^®^ collection, at the Institute for Conservation Research at the San Diego Zoo.

Samples for the RNA sequencing were obtained from five different *A. ventralis* individuals (4 females, 1 male) with an average age of 17 years (±7.4 years) and weight ranging from 234–410 g (median of 262 g) at the School of Veterinary Medicine, University of California, Davis. One liver (sample 336) and four blood samples (sample 335, 140, 341 and 13) were obtained. All birds were part of a research flock and were housed individually in wire cages (61 × 58 × 66 cm) in a room maintained at 23 °C (73.4 °F) with a photoperiod of 12 h. They were fed a pelleted diet (ZuPreem FruitBlend, Premium Nutritional Products, Shawnee, KS, USA) ad libitum and had constant access to water. This study was approved by the University of California, Davis, Institutional Animal Care and Use Committee.

### 2.2. DNA and RNA Extraction

DNA was extracted from whole blood using the Qiagen QIAmp Mini Kit following manufacturer’s protocol. Total RNA was extracted from blood cells and liver tissue using a column method (RNeasy Kit; Qiagen, Hilden, Germany). RNA quality concentration was determined by a fluorometric technique (Qubit, Thermo Fisher Scientific, Waltham, Massachusetts, U.S.A.) and quality was verified by a small fragment analyzer (Bioanalyzer 2100, Agilent, Santa Clara, California, U.S.A.). The globin mRNA content of blood RNA samples (only) was first reduced using a commercial kit (Globin-Zero, Illumina, San Diego, California, U.S.A).

### 2.3. Genome and Transcriptome Sequencing

Genomes of all three species included in this study (*A. vittata*, *A. ventralis* and *A. leucocephala*) were sequenced using the Illumina HiSeq2000 platform. For *A. vittata* one PE (paired-ends) library with 300 bp target IS (insert size) and 2 MP (mate-pairs) libraries with 3 kbp and 8 kbp target IS, respectively, were generated using TruSeq DNA PCR-Free Library Prep Kits. For *A. ventralis* and *A. leucocephala* only one PE library with 300 bp target IS was sequenced (Table S1).

RNA sequencing was performed by the Genomics Core facility at Washington State University (Spokane, WA, USA) using one liver and four blood samples from five different *A. ventralis* individuals. Then, RNA libraries were generated using Illumina TruSeq RNA Library Prep Kit v2 for each sample using 100 ng of input total RNA. Obtained libraries were sequenced using Illumina’s HiSeq2500 machine.

### 2.4. Data QC and Filtering

The initial QC of NGS data was performed using FastQC [33]. Both genomic and transcriptomic reads were filtered in a two-stage process. First, long fragments of Illumina adapters were trimmed using Cookiecutter [34]. Then Trimmomatic v0.36 [35] was used to remove short adapter fragments and perform filtering by quality (Trimmomatic options: ILLUMINACLIP: TRIMMOMATIC_ADAPTERS:2:30:10:1 SLIDINGWINDOW:20:20 MINLEN:50). The resulting output is shown in the Table S1.

### 2.5. Genome and Transcriptome Assembly

The new *A. vittata* genome was assembled from 1 PE library and 2 MP libraries (3 kbp and 8 kbp, see Table S1) followed by scaffolding using *A. ventralis* de novo transcriptome assembly (see Supplementary Figure S1 for the assembly pipeline) and post-assembly filtration. De novo transcriptome assembly for *A. ventralis* was performed using Trinity v2.8.2 [36] from filtered reads for each RNAseq library independently and for merged library including reads from all five libraries generated.

Initial genome contigs were generated from the PE library using Fermi v 1.1 assembler [37]. Then reads from all libraries were aligned to the initial contigs using BWA [38] to estimate actual insert sizes. Only alignments to contigs whose length was equal to or greater than, 3× target IS were used for estimation in order to avoid bias introduced by alignment artifacts. For actual IS see Table S1. At the following step, initial contigs were scaffolded by SSPACE [39] using all read libraries, followed by gap closing with GapCloser [40] using only the PE library. Next, all scaffolds with length of less than 100 bp (i.e., less than read length from PE library) were removed as assembly artifacts. Finally, transcripts from *A. ventralis* transcriptome assembly were aligned to the scaffolds by BLAT [41] and the obtained alignment was used for scaffolding with L_RNA_scaffolder [42]. Finally, all scaffolds of length lower than 1000 bp were discarded. Genome size and actual coverage of PE libraries were estimated using the Jellyfish 2 [43] and KrATER [44] for each species. Assembly integrity was verified using BUSCO v3 and aves_odb9 gene set [45] (Table S2).

### 2.6. Repeat Masking in the A. vittata Genome

Repeat identification in the *A. vitatta* genome was performed de novo from the PE library and the repeat library generated. It was then combined with *aves* repeats from the RepBase [46] and the combined library was used to annotate repeats with RepeatMasker [47,48,49]. Finally, repeats in the *A. vittata* genome were soft-masked using BEDtools [50] for prediction of protein-coding genes.

### 2.7. Annotation of Protein-Coding Genes in the A. vittata Genome

The annotation of protein-coding genes was performed using a combined approach that unifies homology-based, transcriptome-based and de novo predictions. However, de novo predictions were used only to fill gaps and to extend homology- and transcriptome-based predictions. Proteins of three reference species: *Gallus gallus* (Gallus_gallus-5.0 (GCA_000002315.3)), *Melopsittacus undulatus* (melUnd1) and *Taeniopygia guttata* (taeGut3.2.4) were aligned to the *A. vittata* assembly by Exonerate [51] using the *protein2genome* model with a maximum of five hits per protein. The obtained alignments were divided into the top (primary) and secondary hits; the coding sequence (CDS) fragments were cut from each side by 3 bp for the top hits and by 9 bp for the secondary hits. Then, *A. ventralis* RNAseq reads from all libraries were aligned to *A. vittata* genome by STAR [52] and the obtained splice junctions alongside with CDS segments from protein alignments were clustered and supplied as hints to the AUGUSTUS software package [53]. The CDS segments of genes were predicted in a soft-masked *A. vitatta* assembly using chicken gene models. Proteins were extracted from the predicted genes and aligned by HMMER v3.1 [54] and BLAST [55] to the Pfam [56] and Swiss-Prot [57] databases, respectively. Only genes supported by both hints and hits to one of the protein databases were retained; the rest were discarded.

### 2.8. Genome Read Alignment and Variant Calling

Filtered reads of *A. vittata*, *A. ventralis* and *A. leucocephala* were aligned to the assembled *A. vittata* genome using BWA mem with default options, followed by duplicate marking using Picard [58] MarkDuplicates. Next, a mask track was created for each genome using deduplicated alignments and based on coverage. Only regions with coverage of 50–250% (10–50× for *A. ventralis*, 8–40× for *A. leucocephala*, 6–34× for *A. vittata*) of mean coverage were retained unmasked. Then, *HaplotypeCaller* from GATK pipeline [58] was used to call variants. Only the SNPs (Single Nucleotide Polymorphisms) and indels passing hard filters from GATK Best Practice were retained (QD > 2.0, FS < 20.0, MQ > 40.0, MQRankSum > −12.5, ReadPosRankSum > −8.0 for SNPs and QD > 2.0, FS < 20.0, ReadPosRankSum > −20.0 for indels, respectively).

### 2.9. Reference-Assisted Assembly of A.ventralis and A. leucocephala Genomes

In all three species, filtered reads from PE libraries of all three amazons were aligned by the BWA to the previously assembled *A. vittata* reference genome, followed by variant calling using GATK Haplotype caller with extensive filtration in accordance with the GATK best practices. In the case of a heterozygous position being encountered during reference assisted-assembly, the algorithm had to choose between two possible nucleotides. Therefore, several options would be available. First, if both alleles were different from the reference, the algorithm could choose one randomly. Second, if one allele is identical to the one in the reference genome, it would be reasonable to choose the reference allele in the new genome as well. However, the tool for generation of genome sequence in the GATK pipeline [58] chooses the alternative allele by default. Therefore, we had to remove SNPs with reference alleles from the vcf file prior to the reference assisted assembly.

### 2.10. Phylogeny Reconstruction and Divergence Time Estimation

Ortholog identification for the longest proteins corresponding to each predicted gene of *A.vittata, A. ventralis, A. leucocephala* and other species from songbird, parrot and falcon groups was performed using Emapper V 1.0.1 [59] and veNOG subset (dataset for vertebrate orthologs) from the eggNOG database of orthologous groups [59]. Other species included: *Serinus canaria* [60], *Ficedula albicollis* [61], *Parus major* [62], *Zonotrichia albicollis* [63], *Manacus vitellinus* [4], *Cyanistes caeruleus* [64], *Melopsittacus undulatus* [65], *Geospiza fortis* [66], *Taeniopygia guttata* [67], *Aquila chrysaetos* [68] and *Falco peregrinus* [69].

Single-copy orthologs were extracted from the obtained groups and corresponding CDSs (coding sequences) were aligned by codon using PRANK [70], followed by removal of hypervariable regions with Gblocks [71,72]. Obtained alignments were concatenated, being treated as a single partition and used to reconstruct a maximum likelihood tree with RAxML v8.2 [73] under the GTRGAMMA with 1000 bootstrap replications. The reconstructed tree was rooted with *Falconiformes* species (*Falco cherrug*, *Falco peregrinus*) as an outgroup. The resulting tree was drawn using FigTree software [74].

### 2.11. Demographic History Inference

Based on the variation data from the genomes, we estimated population dynamics using the pairwise sequentially Markovian coalescent (PSMC) model [75]. The PSMC approach uses the coalescent model to estimate changes in population size, which allowed us to create a TMRCA (Time to the Most Recent Common Ancestor) distribution across the genome and estimate the effective population size (*N_e_*) in recent evolutionary history (e.g., from 10,000 to 1 million years). Demographic history was inferred separately for each species using a generation time of six years calculated by the captive breeding program for Puerto Rican parrot [76] and mutation rates recently estimated from bird pedigrees available in the literature [77].

### 2.12. Amazona Genome Browser Hub

To provide convenient access to our data, we organized the UCSC genome browser hub [78] containing annotated genomic features of *A. vitatta*, *A. ventralis* and *A. leucocephala* genomes. The features available on the hub include protein-coding genes and RepeatMasker-detected repeats of *A. vitatta,* as well as genomic variants (SNVs and indels) of the three related to *A. vitatta* species as a reference. Also shown are the BigWig tracks [79] that visualize coverage of the reference genome by aligned reads of the three genomes. The track hub file is publicly available online at: http://public.dobzhanskycenter.ru/AmazonaHub/hub.txt. To view the Amazona hub in the UCSC Genome Browser, the user must add the track hub file to the Track Hubs web page: http://genome.ucsc.edu/cgi-bin/hgHubConnect.

## 3. Results

### 3.1. Assembly and Annotation

In this study we used a combination of short read paired-end (PE) read sequences, mate pairs (MP) and transcriptomes to assemble genomes of three closely related amazon species from the Caribbean: *Amazona vittata, A. leucocephala and A. ventralis*. Among these, *A. vittata* was chosen as reference species for genome assembly. Recent demographic history inadvertently shaped the genome of *A. vittata* into an ideal candidate for a de novo sequencing project: its relatively small (1.58 Gb, less than half of the human) genome [80] was expected to be highly invariable due to the recent population bottleneck [26,27]. In addition, this was the only assembly with the long reads: one PE and two MP libraries available for this species. For the two other parrots, only one PE library per species was generated (Table S1).

Genome size estimations, that were based on distributions of 23-mers extracted from the PE libraries, demonstrated similarity between the species with less than 10% difference (Table 1; Figure S2). Moreover, these values were in relative concordance with the publicly available haploid DNA content estimates evaluated using flow cytometry method (C-values) from the Animal Genome Size Database [81]. The observed discrepancy of about 10% (Table 1) is common among all three estimates and may be attributable to the genome regions that have not been covered by sequencing.

Unfortunately, the transcriptomes of the highly endangered status of *A. vittata* were difficult to obtain due to the restrictions at breeding facilities at the Conservation Program of the Puerto Rican Parrot, U.S. Fish and Wildlife Service and the Recovery Program of the Puerto Rican Parrot at the Rıo Abajo State Forest, Departamento de Recursos Naturales y Ambientales de Puerto Rico. Transcriptome sequencing is crucially important for annotation of protein-coding genes, however the de novo assembled transcripts can also be used for the additional scaffolding stage of the genome assembly. In this study, we sequenced transcriptomes for five tissue samples from the Hispaniolan parrot (*A. ventralis*; 4 blood samples and 1 liver sample) using Illumina HiSeq platform. Transcripts were assembled from both independent libraries and merged into one. The characteristics of the assembled transcriptome are presented in Table 2.

The assembly of *A. vittata* genome followed the multistage pipeline described in Section 2.4. of Materials and Methods (Figure S1). To achieve the best results, PE and MP from *A. vittata* were complemented by the transcriptome reads from *A. ventralis* during the transcriptome based scaffolding stage (Figure S1). This approach worked well, probably due to the close evolutionary proximity between the two species and resulted in the total assembly length of 1.45 Gbp (Table 3), which is slightly longer than the 23-mer based estimation, that used PE libraries of the single species (1.42 Gbp; Figure S2). This small increase in the size of genome assembly is likely to be due to the imprecise gap length estimates during scaffolding, in particular at the transcriptome scaffolding stage.

Quality assessment of our assembly was performed using BUSCOv3 with the *aves_odb9* gene set [45]. Out of the 4915 Benchmarking Universal Single-Copy Orthologs (a conservative gene set or BUSCOs), 87.4% were found as a single-copies, 6.4% as fragmented and 6.2% were not found. Therefore, despite the relatively low PE library coverage common to all three parrot datasets (Table 1), the contig N50 of 101 kbp and the relatively high BUSCO score point to the high integrity of this assembly, sufficient for protein-coding gene prediction. The total number of scaffolds in our assembly, even after filtration of very short scaffolds (shorter than 1000 bp), was relatively high, at more than 62 k (Table 3). However, this is a known issue for the short-read based assemblies with few mate-pair libraries sequenced and it was observed in the earlier assembly as well [27].

Repeat masking is strictly necessary for prediction of protein-coding genes, as interspersed repeats often include mobile elements with ORFs. Unfortunately, the most commonly used database of repetitive elements RepBase still includes a very small number of avian repeats (less than 500). To address this problem, we assembled repeats de novo from the PE library and combined the results with the RepBase *aves* library [46,47,48,49]. Subsequently, 7.57% of the genomic sequences in this study were identified as repeats (Table 4). The most common repeat class appears to be L3/CR1 LTRs, which comprises almost 1/3 of all repeats (2.47% of the genome, Table 4). At the same time, more than half of all repeats still fall into the unclassified category (3.92%, Table 4).

Prediction of protein-coding genes was performed in accordance with the hybrid pipeline (described in Materials and Methods 2.7) using homology (Pfam [56] and Swiss-Prot [57,58] databases), transcriptome (*A. ventralis* RNAseq reads) and de novo predictions. As a reference for homology-based transfer, we have chosen protein sequences of three species: *Gallus gallus* (chicken)*, Taeniopygia guttata* (zebra finch) and *Melopsittacus undulatus* (budgerigar). Chicken and zebra finch were selected as avian species, with the best available chromosome-level assembly and annotation, based on extensive usage of RNAseq. Meanwhile, the budgerigar genome was included because it is the highest quality parrot genome available, which makes it the best currently available closely related species with sufficient assembly and annotation data.

As a result, a total of 19,669 genes have been predicted for the current version of the *A. vittata* genome. This number is somewhat higher than the comparable numbers in genomes of other birds (Table 5). However, the number of genes with the longest protein containing less than 100 amino-acids is 4–9× higher in our annotation than in annotations of other bird genomes (Table 5). This probably reflects the higher fragmentation level of the assembly. All of the assembly data from this study is available in the form of the browser hub. The track hub file is publicly available by the following link: http://public.dobzhanskycenter.ru/AmazonaHub/hub.txt.

### 3.2. Genome-Wide Heterozygosity

Given the fragmented nature (N50 101.028 kbp, Table 3) of our assembly we were unable to use the most common window-based approach for heterozygosity-level assessment. Instead we calculated a simple metric of whole genome heterozygosity: counting and dividing the number of heterozygous SNP by the unmasked genome length. As expected, the *A. vittata* genome had the lowest level of heterozygosity, with a mean density of 0.96 heterozygous SNPs/kbp. *A. ventralis* and *A. lecucocephala* showed higher heterozygosity 1.6–1.7 SNPs/kbp (Table S6).

### 3.3. Phylogenetic and Demographic Analysis

**Figure 2:**
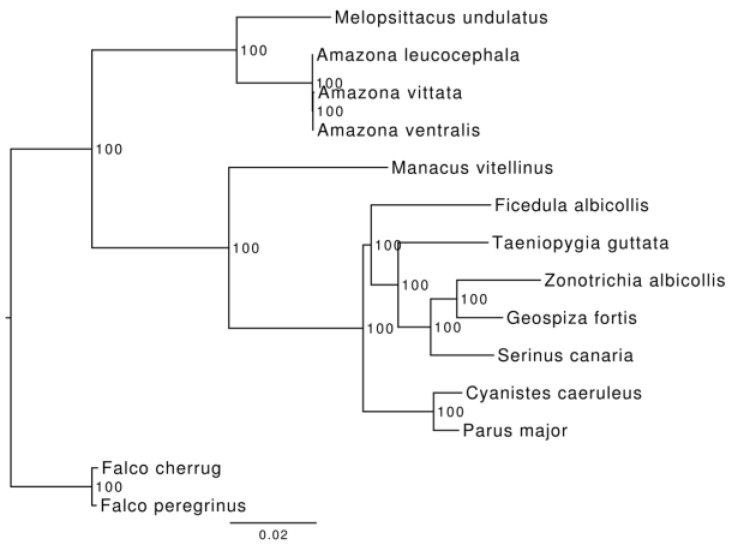
Reconstructed phylogenetic tree for 14 species. Reconstruction was performed using RAxML 8 [63] with falcons (*Falco cherrug* and *F. peregrinus*) as an outgroup. *A. vittata* and *A. ventralis* form a monophyletic group, with *A. leucocephala* as their sister taxon.

In order to resolve evolutionary relationships between the species in this study, we performed in the wider context of bird phylogeny. In addition to the genome data for the three amazon parrots from this study, we chose 11 additional avian genomes. We included two species of falcons (*Falconiformes*), eight species of passerine birds (*Passeriformes*) and one additional parrot (*Psittaciformes*) with assembly and annotation based on extensive usage of RNAseq (see Materials and Methods). The tree was calculated using filtered alignment of 4135 single-copy orthologs in RaxML v.8 with 1000 bootstrap replicates. The two falcon species (*Falco cherrug* and *F. peregrinus*) were placed in an outgroup. All nodes in the resulting tree had the highest 100% bootstrap support suggesting high stability of the tree to the noise in input data. According to our reconstruction *A. vittata* and *A. ventralis* form a monophyletic group with *A. leucocephala* as a sister species (Figure 2).

**Figure 3:**
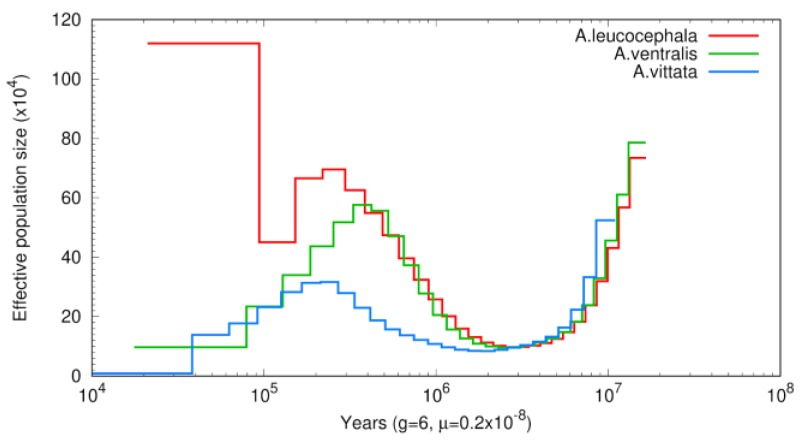
Population history of the three *Amazona* species. For all of them, *A. vittata* genome was used as a reference. Generation times were calculated by the captive breeding program for Puerto Rican parrot [76] and mutation rates recently estimated from bird pedigrees available in the literature [77]. Trajectories of all three species suggest an initial founder effect that may be attributed to parrot dispersal from Central America between 2 and 3 MYA.

We also attempted to estimate population dynamics using the pairwise sequentially Markovian coalescent (PSMC) model. The resulting estimate is based on the assumption of a generation time of six years calculated by the captive breeding program for the Puerto Rican parrot [76] and mutation rates recently estimated from bird pedigrees available in the literature [77] and provides the assessment of these species’ effective population sizes (*N_e_*) in recent evolutionary history (Figure 3). This analysis indicates that the first split between the three species of amazons occurred at least 2 MYA. This date may be suggesting the time of the initial dispersal of the ancestral population of parrots from Central America (Figure 3).

## 4. Discussion

Publicly available genome assemblies and gene annotations for the three Caribbean parrots are the major result of this study. In a combination approach that used both genome and transcriptome sequences, we were able to obtain enough coverage to allow for the identification of the major types of repeats, as well as locations of the protein coding genes (Table 4 and Table 5), with high confidence: 90% of BUSCO genes were found as complete, single copy or duplicated and only 6.4% as fragmented; Table S2. Still, the number of genes (19,669) is somewhat higher than the respective numbers estimated for other birds (Table 5). While BUSCO assessments elevate the confidence that this increase can be correlated with the increase in genome size, we cannot rule out the possibility that the higher fragmentation level of our assemblies has contributed as well. Specifically, the number of genes with longest protein shorter than 100 amino-acids is 4–9 times higher in our annotation than in annotations of other bird genomes (Table 5). Nevertheless, these assemblies were sufficient to produce the first estimates of genome wide heterozygosity (Table S4) and allowed inferences of the phylogeny based on the genome wide data (Figure 2), as well as estimates of demographic histories, in these three island species for the first time (Figure 3).

This study reinforces the observation that the *Amazona* parrots have slightly larger genomes than other parrots on average. The Animal Genome Size Database [81] features haploid DNA content (C-values) for 56 genomes of *Psittaciformes*, of which 16 belong to the genus *Amazona*. The average genome size for the Amazon parrots is 1.58 *pg* (±0.09), while the rest of the parrots have significantly (*p* < 0.0001) smaller genomes of 1.35 *pg* (±0.11). Our estimates were based on distributions of 23-mers extracted from PE libraries (Figure S2, Table 1). Therefore, current genome size estimations for the three Caribbean parrots in this study provide an independent confirmation and demonstrated remarkable similarity with the publicly available C-values. The 10% discrepancy in genome size estimates between the two methods (Table 1) could be attributed to the genomic regions which have not been covered by sequencing.

Only 7.57% of the parrot genomic sequences in this study were identified as repeats (Table 4). Such a small fraction is not unusual for birds. In fact, birds have the least number of repeated elements in their genomes compared to any other group of tetrapods, comprising only 4–10% of the total genome size (compared to the 34–52% in mammals) [4,80] and an up to two-fold genome contraction had occurred before the divergence of birds from a theropod ancestor [82,83,84]. However, since repeats are more difficult to assemble and a higher proportion of them would be omitted in comparison with the rest of the sequence, the reduced number of repeats we found could also be explained by the high percentage of gaps (~24%) in the present genome assembly.

There is a possibility that the large genome sizes in Amazons could be attributed to expansion of one of the transposable element classes. Unfortunately, because of the high gap proportion in the current genomes, it is difficult to determine which of these elements could be the culprit with high confidence. The most common of repeats we have identified are the Chicken repeat 1 (CR1) elements that make up 2.47% of the Amazona genome (Table 4). CR1 elements belong to the non-long repeat class of retrotransposons and are subdivided into at least six distinct subfamilies, comprising sequences of about 300 bp long, all of which share substantial sequence similarity. CR1-like elements were found in various genomes from invertebrates to mammals, suggesting their importance for genome structure and/or function [85]. However, it is too early to arrive at any conclusion, since the majority of the repetitive content is either not represented in the assembly (because repeats are more difficult to assemble and place) or has been labelled as “unclassified” (3.92% of the genome; Table 4).

There were two main reasons why we could not calculate the timing of speciation using the divergence time analysis given our data. First, we noted a significant difference in cumulative branch length (especially for *Passeriformes*) and suspected existence of substantial differences in the mutation rates that would bias our estimates. Second, the current paleontological record is lacking fossil calibrations inside *Psittaciformes.* The only calibration point within the parrot clade for a split between *Melopsittacus undulatus* and Amazons (22.5 MYA) is in fact a derived second level molecular calibration point. Fossil-based calibration points within the passerine clade (split between *Manacus/Taeniopygia/Geospiza* at 13.6–16.3 MYA and *Taeniopygia/Geospiza* at 7.2–11.6 [2]) are not useful for timing parrot phylogenies because of the mentioned issue involving mutation rate. The split between *Psittaciformes*/*Passeriformes* (53.5–65 MYA) [86] is too old to be helpful for dating of the relatively recent split between *Amazona* species. Hopefully, paleontological discoveries of new fossils of ancient parrots combined with the rapid advances in genome sequencing and analysis will soon bridge this gap. All mentioned fossil calibrations are listed in a supplementary table (Table S3).

In this study we present an early attempt to estimate genome wide heterozygosity given our data. This early estimate can be further evaluated and discussed in conjunction with the effects for life-history, morphological and physiological traits [87,88,89]. Species that are endangered and/or threatened taxa generally display lower heterozygosity than related unthreatened taxa [90]. However, we can already use these preliminary values to address certain theoretical questions in conservation genetics. For instance, we used the heterozygote estimates in context with the hypothesis that genetic diversity should be positively correlated among islands [91]. This hypothesis is based on two of the most prominent theories of island diversity, MacArthur and Wilson’s [6] theory of island biogeography and Sewall Wright’s [92] island model of population genetics [93]. While we have observed a connection between island size and heterozygosity (Figure S3; r^2^ > 0.99), more island species are needed for a better evaluation of this hypothesis (Figure S3). The heteroygosity estimates for SNPs based on the genome sequences indicate that the Puerto Rican parrot has the lowest heterozygosity (0.96 SNPs/kbp), with almost half of that in the other two species and is similar to the number reported for another endangered parrot: kea (*Nestor notabilis*; 0.91 SNPs/kbp). At the same time, two earlier investigated critically endangered/vulnerable avian species, the white-tailed eagle (0.4 SNPs/kbp) and the dalmatian pelican (0.6 SNPs/kbp), have lower heterozygosity values [94] (Table S4).

In conclusion, new data generated on three Caribbean amazons has contributed to the body of knowledge on parrot genomics and conservation genetics and in combination with other genomes it will allow for future analyses that will provide valuable insights into the evolution of functional elements in the genomes of these parrot species.

## Figures and Tables

**Table 1:** Genome size estimates for the three Amazon parrot species in this study.

Parrot Species	PE Library Coverage	Genome Size (Gbp)	C-Value (pg)
*Amazona vitatta*	14×	1.42	1.58
*A. ventralis*	22×	1.42	1.62–1.65
*A. leucocephala*	16×	1.54	1.58–1.65

23-mer based estimate based on sequencing data in this study; C-values are from the Animal Genome Size Database [81].

**Table 2:** Statistics for RNAseq libraries and assembled transcripts.

Library ID	Tissue	Read Pairs (Millions)	Bases (Gbp)	Assembled Transcripts
Parrot13	Blood	54.2	10.7	314,505
Parrot140	Blood	64.9	12.7	378,318
Parrot335	Blood	47.3	9.3	306,123
Parrot341	Blood	54.2	10.7	326,706
Parrot_336	Liver	69.6	13.7	210,549
**Merged**	**-**	**290.3**	**57.1**	**680,785**

**Table 3:** Metrics for the *Amazona vittata* de novo genome assembly.

	N50 (kbp)	L50 (kbp)	Longest Contig (Mb)	Number of Ns Mb	Number of Scaffolds	Assembled Genome Length (Gbp)
*Amazona vittata*	101.028	3057	1.885	367.97	62,777	1.447

**Table 4:** Repeat content of the *Amazona vittata* genome annotated by RepeatMasker [47,48,49] using generated de novo library combined with *Aves* repeats from RepBase [46].

Class	Number of Repeats	Total Length (bp)	Percentage of the Genome (%)
**Total repeats:**		**107,498,949**	**7.43%**
**SINEs**	**6995**	**895,617**	**0.06%**
ALUs	0	0	0.00%
MIRs	3794	414,542	0.03%
**LINEs:**	**147,387**	**36,382,264**	**2.51%**
LINE1	80	19,168	0.00%
LINE2	2175	474,752	0.03%
L3/CR1	144,820	35,707,674	2.47%
**LTR elements**	**34,688**	**10,514,590**	**0.73%**
ERVL	31,743	9,376,828	0.65%
ERVL-MaLRs	0	0	0.00%
ERV_classI	1826	779,161	0.05%
ERV_classII	939	326,675	0.02%
**DNA elements:**	**19,273**	**3,034,179**	**0.21%**
hAT-Charlie	201	57,997	0.00%
TcMar-Tigger	273	49,514	0.00%
**Unclassified:**	**345,805**	**56,672,299**	**3.92%**
**Small RNA:**	**2066**	**272,961**	**0.02%**
**Satellites:**	**3155**	**513,361**	**0.04%**
**Simple repeats:**	**8207**	**1,700,409**	**0.12%**
**Low complexity:**	**256**	**63,286**	**0.00%**

**Table 5:** Statistics for protein-coding genes of species used in the current analysis. Among the three *Amazona* species used, only the *A. vittata* is listed, since as for *A. leucocephala* and *A. ventralis* only reference-assisted assemblies were performed, so that identical (or almost identical) gene counts are reported for these species.

Species	N of Genes	N of Genes with Longest Protein <100 aa	N Genes Assigned to the EggNOG Clusters	Genome Size (pg) *
*Cyanistes caeruleus*	16,519	503	16,030	1.47
*Falco cherrug*	14,694	302	14,607	-
*Falco peregrinus*	14,859	307	14,771	1.45
*Ficedula albicollis*	15,400	360	14,952	-
*Geospiza fortis*	14,182	327	14,101	-
*Manacus vitellinus*	16,312	362	16,086	-
*Melopsittacus undullatus*	14,255	315	14,192	1.02–1.37
*Parus major*	15,251	285	14,795	1.51
*Serinus canaria*	15,582	455	15,194	1.48–1.62
*Taenopygia guttata*	16,368	494	16,202	1.25
*Zonotrichia albicollis*	14,374	314	14,018	1.33–1.58
*Amazona vittata*	19,669	2339	18,488	1.58

* C-values are from the Genome Size Database [81].

## Supplementary Materials

All the data has been uploaded to NCBI: Bioproject accession PRJNA496322; Genome submission ID: SUB4629817. The following are available online at http://www.mdpi.com/2073-4425/10/1/54/s1, **Figure S1.** Pipeline used to assemble *A. vitatta* genome. One PE and two MP *A. vitatta* genome libraries were used to generate preliminary scaffolds complemented by additional scaffolding step using five *A. ventralis* RNAseq libraries. **Figure S2.** Distributions of 23-mers from K-mer distributions PE libraries of three parrot species. Corresponding genome size estimation present on figure and also in Table 1. **Figure S3**. Connection between heterozygosity and areal size for three *Amazona* species. **Table S1.** The sequencing outputs for each genome used in the current assemblies. Abbreviations: paired-end (PE) mate pairs (MP); **Table S2.** BUSCO scores for all steps of assembly of *A. vittata* genome. Assembly evaluation was performed using BUSCO v3 and Avian dataset (Simão et al., 2015). **Table S3.** Available fossil-based calibrations for speciation time dating within Psittaciformes and Passeriformes clades. **Table S4.** Mean heterozygosity in the genomes of three *Amazona* species compared with the values reported earlier for other avian species.

## References

[1] Zhang G., Jarvis E.D., Gilbert M.T.P. (2014). A flock of Genomes. Science.

[2] Zhang G. (2015). Genomics: Bird sequencing project takes off. Nature.

[3] Jarvis E.D., Mirarab S., Aberer A.J., Li B., Houde P., Li C., Ho S.Y.W., Faircloth B.C., Nabholz B., Howard J.T. (2014). Whole-genome analyses resolve early branches in the tree of life of modern birds. Science.

[4] Zhang G., Li C., Li Q., Li B., Larkin D.M., Lee C., Storz J.F., Antunes A., Greenwold M.J., Meredith R.W. (2014). Comparative genomics reveals insights into avian genome evolution and adaptation. Science.

[5] Grant P.R., Grant B.R. (2002). Adaptive radiation of Darwin’s finches. Am. Sci..

[6] MacArthur R.H., Wilson E.O. (1967). The Theory of Island Biogeography.

[7] Darwin C. (1845). Journal of Researches into the Natural History and Geology of the Countries Visited during the Voyage of H.M.S. Beagle Round the World, under the Command of Capt. Fitz Roy, R.N. (Voyage on the Beagle).

[8] Whittaker R.J., Fernández-Palacios J.M., Matthews T.J., Borregaard M.K., Triantis K.A. (2017). Island biogeography: Taking the long view of nature’s laboratories. Science.

[9] O’Brien S.J. (2012). Genome empowerment for the Puerto Rican parrot—Amazona vittata. Gigascience.

[10] Snyder N., Wiley J.W., Kepler C.B. (1987). The Parrots of Luquillo: Natural History and Conservation of the Puerto Rican Parrot.

[11] Lack D. (1976). Island Biology Illustrated by the Land Birds of Jamaica.

[12] Russello M.A., Amato G. (2004). A molecular phylogeny of Amazona: Implications for Neotropical parrot biogeography, taxonomy, and conservation. Mol. Phylogenet. Evol..

[13] Blanco G., Hiraldo F., Rojas A., Dénes F.V., Tella J.L. (2015). Parrots as key multilinkers in ecosystem structure and functioning. Ecol. Evol..

[14] Blanco G., Hiraldo F., Tella J.L. (2018). Ecological functions of parrots: An integrative perspective from plant life cycle to ecosystem functioning. Emu Aust. Ornithol..

[15] Aslan C.E., Zavaleta E.S., Croll D., Tershy B. (2012). Effects of Native and Non-Native Vertebrate Mutualists on Plants. Conserv. Biol..

[16] Anderson S.H., Kelly D., Ladley J.J., Molloy S., Terry J. (2011). Cascading effects of bird functional extinction reduce pollination and plant density. Science.

[17] Cracraft J. (2001). Avian evolution, Gondwana biogeography and the Cretaceous-Tertiary mass extinction event. Proc. R. Soc. B Biol. Sci..

[18] Wright T.F., Schirtzinger E.E., Matsumoto T., Eberhard J.R., Graves G.R., Sanchez J.J., Capelli S., Müller H., Scharpegge J., Chambers G.K. (2008). A multilocus molecular phylogeny of the parrots (Psittaciformes): Support for a gondwanan origin during the cretaceous. Mol. Biol. Evol..

[19] Rheindt F.E., Christidis L., Kuhn S., de Kloet S., Norman J.A., Fidler A. (2014). The timing of diversification within the most divergent parrot clade. J. Avian Biol..

[20] Prum R.O., Berv J.S., Dornburg A., Field D.J., Townsend J.P., Moriarty Lemmon E., Lemmon A.R. (2015). A Comprehensive Phylogeny of Birds (Aves) using Targeted Next Generation DNA Sequencing Online Data and Software Archive. Nature.

[21] Claramunt S., Cracraft J. (2015). A new time tree reveals Earth history’s imprint on the evolution of modern birds. Sci. Adv..

[22] Mayr G. (2014). The origins of crown group birds: Molecules and fossils. Palaeontology.

[23] Mayr G. (2009). Paleogene Fossil Birds.

[24] Ottens-Wainright P., Halanych K.M., Eberhard J.R., Burke R.I., Wiley J.W., Gnam R.S., Aquilera X.G. (2004). Independent geographic origin of the genus Amazona in the West Indies. J. Caribb. Ornithol..

[25] Avise J.C. (2000). Phylogeography: The History and Formation of Species.

[26] Kolchanova S. (2018). Molecular Phylogeny and Evolution of Amazon Parrots in the Greater Antilles.

[27] Oleksyk T.K., Pombert J.-F., Siu D., Mazo-Vargas A., Ramos B., Guiblet W., Afanador Y., Ruiz-Rodriguez C.T., Nickerson M.L., Logue D.M. (2012). A locally funded Puerto Rican parrot (Amazona vittata) genome sequencing project increases avian data and advances young researcher education. Gigascience.

[28] Brinkley D. (2009). The Wilderness Warrior: Theodore Roosevelt and the Crusade for America.

[29] Afanador Y., Velez-Valentín J., Valentín de la Rosa R., Martínez-Cruzado J.C., vonHoldt B., Oleksyk K.T. (2014). Isolation and characterization of microsatellite loci in the critically endangered Puerto Rican parrot (Amazona vittata). Conserv. Genet. Resour..

[30] Brock M.K., White B.N. (1992). Application of DNA fingerprinting to the recovery program of the endangered Puerto Rican parrot. Proc. Natl. Acad. Sci. USA.

[31] Allendorf F.W., Hohenlohe P.A., Luikart G. (2010). Genomics and the future of conservation genetics. Nat. Rev. Genet..

[32] Ouborg N.J., Pertoldi C., Loeschcke V., Bijlsma R.K., Hedrick P.W. (2010). Conservation genetics in transition to conservation genomics. Trends Genet..

[33] Andrews S. (2010). FASTQC. A Quality Control Tool for High Throughput Sequence Data. http://www.bioinformatics.babraham.ac.uk/projects/fastqc/.

[34] Starostina E., Tamazian G., Dobrynin P., O’Brien S., Komissarov A. (2015). Cookiecutter: A tool for kmer-based read filtering and extraction. bioRxiv.

[35] Bolger A.M., Lohse M., Usadel B. (2014). Trimmomatic: A flexible trimmer for Illumina sequence data. Bioinformatics.

[36] Grabherr M.G., Haas B.J., Yassour M., Levin J.Z., Thompson D.A., Amit I., Adiconis X., Fan L., Raychowdhury R., Zeng Q. (2011). Trinity: Recontructing a full-length transcriptome assembly without a genome from RNA-Seq data. Nat. Biotechnol..

[37] Grigorev K., Kliver S., Dobrynin P., Komissarov A., Wolfsberger W., Krasheninnikova K., Afanador-Hernández Y.M., Paulino L.A., Carreras R., Rodríguez L.E. (2018). Innovative assembly strategy contributes to the understanding of evolution and conservation genetics of the critically endangered *Solenodon paradoxus* from the island of Hispaniola. GigaScience.

[38] Li H., Durbin R. (2009). Fast and accurate short read alignment with Burrows-Wheeler transform. Bioinformatics.

[39] Boetzer M., Henkel C.V., Jansen H.J., Butler D., Pirovano W. (2011). Scaffolding pre-assembled contigs using SSPACE. Bioinformatics.

[40] Luo R., Liu B., Xie Y., Li Z., Huang W., Yuan J., He G., Chen Y., Pan Q., Liu Y. (2012). SOAPdenovo2: An empirically improved memory-efficient short-read de novo assembler. Gigascience.

[41] Kent W.J. (2002). BLAT—The BLAST-like alignment tool. Genome Res..

[42] Xue W., Li J.T., Zhu Y.P., Hou G.Y., Kong X.F., Kuang Y.Y., Sun X.W. (2013). L_RNA_scaffolder: Scaffolding genomes with transcripts. BMC Genom..

[43] Marçais G., Kingsford C. (2011). A fast, lock-free approach for efficient parallel counting of occurrences of k-mers. Bioinformatics.

[44] Kliver S. KRATeR. https://github.com/mahajrod/KrATER.

[45] Simão F.A., Waterhouse R.M., Ioannidis P., Kriventseva E.V., Zdobnov E.M. (2015). BUSCO: Assessing genome assembly and annotation completeness with single-copy orthologs. Bioinformatics.

[46] Bao W., Kojima K.K., Kohany O. (2015). Repbase Update, a database of repetitive elements in eukaryotic genomes. Mob. DNA.

[47] Smit A., Hubley R., Green P. RepeatMasker Open-4.0. 2013–2018. http://www.repeatmasker.org.

[48] Smit A., Hubley R. RepeatModeler Open-1.0. https://github.com/rmhubley/RepeatModeler/blob/master/README.

[49] Tarailo-Graovac M., Chen N. (2009). Using RepeatMasker to identify repetitive elements in genomic sequences. Curr. Protoc. Bioinform..

[50] Quinlan A.R., Hall I.M. (2010). BEDTools: A flexible suite of utilities for comparing genomic features. Bioinformatics.

[51] Slater G.S.C., Birney E. (2005). Automated generation of heuristics for biological sequence comparison. BMC Bioinformatics.

[52] Dobin A., Davis C.A., Schlesinger F., Drenkow J., Zaleski C., Jha S., Batut P., Chaisson M., Gingeras T.R. (2013). STAR: Ultrafast universal RNA-seq aligner. Bioinformatics.

[53] Stanke M., Keller O., Gunduz I., Hayes A., Waack S., Morgenstern B. (2006). AUGUSTUS: Ab initio prediction of alternative transcripts. Nucleic Acids Res..

[54] Johnson L.S., Eddy S.R., Portugaly E. (2010). Hidden Markov model speed heuristic and iterative HMM search procedure. BMC Bioinform..

[55] Altschul S.F., Gish W., Miller W., Myers E.W., Lipman D.J. (1990). Basic local alignment search tool. J. Mol. Biol..

[56] Bateman A., Coin L., Durbin R., Finn R.D., Hollich V., Griffiths-Jones S., Khanna A., Marshall M., Moxon S., Sonnhammer E.L.L. (2004). The Pfam protein families database. Nucleic Acids Res..

[57] Bateman A., Martin M.J., O’Donovan C., Magrane M., Alpi E., Antunes R., Bely B., Bingley M., Bonilla C., Britto R. (2017). UniProt: The universal protein knowledgebase. Nucleic Acids Res..

[58] Van der Auwera G.A., Carneiro M.O., Hartl C., Poplin R., del Angel G., Levy-Moonshine A., Jordan T., Shakir K., Roazen D., Thibault J. (2013). From fastQ data to high-confidence variant calls: The genome analysis toolkit best practices pipeline. Curr. Protoc. Bioinform..

[59] Huerta-Cepas J., Szklarczyk D., Forslund K., Cook H., Heller D., Walter M.C., Rattei T., Mende D.R., Sunagawa S., Kuhn M. (2015). eggNOG 4.5: A hierarchical orthology framework with improved functional annotations for eukaryotic, prokaryotic and viral sequences. Nucleic Acids Res..

[60] Frankl-Vilches C., Kuhl H., Werber M., Klages S., Kerick M., Bakker A., de Oliveira E.H.C., Reusch C., Capuano F., Vowinckel J. (2015). Using the canary genome to decipher the evolution of hormone-sensitive gene regulation in seasonal singing birds. Genome Biol..

[61] Ellegren H., Smeds L., Burri R., Olason P.I., Backström N., Kawakami T., Künstner A., Mäkinen H., Nadachowska-Brzyska K., Qvarnström A. (2012). The genomic landscape of species divergence in Ficedula flycatchers. Nature.

[62] Laine V.N., Gossmann T.I., Schachtschneider K.M., Garroway C.J., Madsen O., Verhoeven K.J.F., de Jager V., Megens H.-J., Warren W.C., Minx P. (2016). Evolutionary signals of selection on cognition from the great tit genome and methylome. Nat. Commun..

[63] Balakrishnan C.N., Mukai M., Gonser R.A., Wingfield J.C., London S.E., Tuttle E.M., Clayton D.F. (2014). Brain transcriptome sequencing and assembly of three songbird model systems for the study of social behavior. PeerJ.

[64] Mueller J.C., Kuhl H., Timmermann B., Kempenaers B. (2016). Characterization of the genome and transcriptome of the blue tit Cyanistes caeruleus: Polymorphisms, sex-biased expression and selection signals. Mol. Ecol. Resour..

[65] Ganapathy G., Howard J.T., Ward J.M., Li J., Li B., Li Y., Xiong Y., Zhang Y., Zhou S., Schwartz D.C. (2014). High-coverage sequencing and annotated assemblies of the budgerigar genome. Gigascience.

[66] Zhang G., Parker P., Li B., Li H., Wang J., Parker P., Li B., Li H., Wang J. (2012). The genome of Darwin’s Finch (Geospiza fortis). GigaDB.

[67] Warren W.C., Clayton D.F., Ellegren H., Arnold A.P., Hillier L.W., Künstner A., Searle S., White S., Vilella A.J., Fairley S. (2010). The genome of a songbird. Nature.

[68] Doyle J.M., Katzner T.E., Bloom P.H., Ji Y., Wijayawardena B.K., DeWoody J.A. (2014). The Genome Sequence of a Widespread Apex Predator, the Golden Eagle (*Aquila chrysaetos*). PLoS ONE.

[69] Zhan X., Pan S., Wang J., Dixon A., He J., Muller M.G., Ni P., Hu L., Liu Y., Hou H. (2013). Peregrine and saker falcon genome sequences provide insights into evolution of a predatory lifestyle. Nat. Genet..

[70] Löytynoja A., Goldman N. (2010). webPRANK: A phylogeny-aware multiple sequence aligner with interactive alignment browser. BMC Bioinform..

[71] Talavera G., Castresana J. (2007). Improvement of phylogenies after removing divergent and ambiguously aligned blocks from protein sequence alignments. Syst. Biol..

[72] Castresana J. (2000). Selection of conserved blocks from multiple alignments for their use in phylogenetic analysis. Mol. Biol. Evol..

[73] Stamatakis A. (2014). RAxML version 8: A tool for phylogenetic analysis and post-analysis of large phylogenies. Bioinformatics.

[74] Rambaut A. (2016). FigTree. http://tree.bio.ed.ac.uk/software/figtree/.

[75] Li H., Durbin R. (2011). Inference of human population history from individual whole-genome sequences. Nature.

[76] Earnhardt J., Vélez-Valentín J., Valentin R., Long S., Lynch C., Schowe K. (2014). The Puerto Rican parrot reintroduction program: Sustainable management of the aviary population. Zoo Biol..

[77] Smeds L., Qvarnström A., Ellegren H. (2016). Direct estimate of the rate of germline mutation in a bird. Genome Res..

[78] Raney B.J., Dreszer T.R., Barber G.P., Clawson H., Fujita P.A., Wang T., Nguyen N., Paten B., Zweig A.S., Karolchik D. (2014). Track data hubs enable visualization of user-defined genome-wide annotations on the UCSC Genome Browser. Bioinformatics.

[79] Kent W.J., Zweig A.S., Barber G., Hinrichs A.S., Karolchik D. (2010). BigWig and BigBed: Enabling browsing of large distributed datasets. Bioinformatics.

[80] Tiersch T.R., Wachtel S.S. (1991). On the evolution of genome size of birds. J. Hered..

[81] Gregory T. Animal Genome Size Database. http://www.genomesize.com.

[82] Volfovsky N., Oleksyk T.K., Cruz K.C., Truelove A.L., Stephens R.M., Smith M.W. (2009). Chimpanzee chromosome 23 vs. human 22: Genomic insertion, deletion and ancestral indel polymorphisms. BMC Genom..

[83] Organ C.L., Shedlock A.M., Meade A., Pagel M., Edwards S.V. (2007). Origin of avian genome size and structure in non-avian dinosaurs. Nature.

[84] Kapusta A., Suh A., Feschotte C. (2017). Dynamics of genome size evolution in birds and mammals. Proc. Natl. Acad. Sci. USA.

[85] Coullin P., Bed’Hom B., Candelier J.J., Vettese D., Maucolin S., Moulin S., Galkina S.A., Bernheim A., Volobouev V. (2005). Cytogenetic repartition of chicken CR1 sequences evidenced by PRINS in Galliformes and some other birds. Chromosom. Res..

[86] Ksepka D., Clarke J. (2015). Phylogenetically vetted and stratigraphically constrained fossil calibrations within Aves. Palaeontol. Electron..

[87] DeWoody Y.D., DeWoody J.A. (2005). On the estimation of genome-wide heterozygosity using molecular markers. J. Hered..

[88] Coltman D.W., Slate J. (2003). Microsatellite measures of inbreeding: A meta-analysis. Evolution.

[89] Chapman J.R., Nakagawa S., Coltman D.W., Slate J., Sheldon B.C. (2009). A quantitative review of heterozygosity-fitness correlations in animal populations. Mol. Ecol..

[90] Spielman D., Brook B.W., Frankham R. (2004). Most species are not driven to extinction before genetic factors impact them. Proc. Natl. Acad. Sci. USA.

[91] Vellend M. (2003). Island Biogeography of Genes and Species. Am. Nat..

[92] Wright S. (1940). Breeding Structure of Populations in Relation to Speciation. Am. Nat..

[93] Fleming T.H. (2010). The theory of island biogeography at age 40. Evolution.

[94] Li S., Li B., Cheng C., Xiong Z., Liu Q., Lai J., Carey H.V., Zhang Q., Zheng H., Wei S. (2014). Genomic signatures of near-extinction and rebirth of the crested ibis and other endangered bird species. Genome Biol..

