# Supplementary material for: Genomes of Three Closely Related Caribbean Amazons Provide Insight for Species History and Conservation
Source: Genes (Basel). 2019 Jan 16;10(1):54. doi: 10.3390/genes10010054 (PMC6356210; doi:10.3390/genes10010054)
Supplement: Supplementary file 1 [file genes-10-00054-s001.pdf]

genes

Supplementary Materials

Genomes of three closely related Caribbean Amazons provide insight for species history and conservation

Sofiia Kolchanova [1,2,3], Sergei Kliver [3,*], Aleksei Komissarov [3], Pavel Dobrinin [3], Gaik Tamazian [3], Kirill Grigorev [1,4], Walter W. Wolfsberger [1,5,6], Audrey J. Majeske [1], Jafet Velez-Valentın [8], Ricardo Valentın de la Rosa [9], Joanne R. Paul-Murphy [10], David Sanchez-Migallon Guzman [10], Michael H. Court [11], Juan L. Rodriguez-Flores [4], Juan Carlos Martínez-Cruzado [1] and Taras K. Oleksyk [1,5,6,*]

1 Department of Biology, University of Puerto Rico at Mayaguez, Mayaguez, PR 00680, USA; sofiia.kolchanova@upr.edu (S.K.); kig2007@med.cornell.edu (K.G.); wwolfsberger@oakland.edu (W.W.W.); audrey.majeske@upr.edu (A.J.M.); juancarlos.martinez@upr.edu (J.C.M.-C.)
2 Department of Biology, University of Konstanz, 78464 Konstanz, Germany
3 Theodosius Dobzhansky Center for Genome Bioinformatics, St. Petersburg State University, 199034 St. Petersburg, Russia; ad3002@gmail.com (A.K.); pdobrynin@gmail.com (P.D.); gaik.tamazian@gmail.com (G.T)
4 Department of Genetic Medicine, Weill Cornell Medical College, New York, NY 10021, USA; jur2014@med.cornell.edu
5 Department of Biological Sciences, Oakland University, 118 Library Drive, Rochester, MI 48309, USA
6 Department of Biological Sciences, Uzhhorod National University, 88000 Uzhhorod, Ukraine
7 Beaumont BioBank, William Beaumont Hospital, Royal Oak, MI 48073, USA;
8 Conservation Program of the Puerto Rican Parrot, U.S. Fish and Wildlife Service, Rio Grande, PR 00745, USA; jafet_velez@fws.gov
9 The Recovery Program of the Puerto Rican Parrot at the Rio Abajo State Forest, Departamento de Recursos Naturales y Ambientales de Puerto Rico, Arecibo, PR 00613, USA; el.cotorro.electrico@gmail.com
10 Department of Medicine and Epidemiology, School of Veterinary Medicine, University of California Davis, Davis, CA 95616, USA; paulmurphy@ucdavis.edu (J.R.P.-M.); guzman@ucdavis.edu (D.S.-M.G.)
11 Program in Individualized Medicine (PrIMe), Pharmacogenomics Laboratory, Department of Veterinary Clinical Sciences, College of Veterinary Medicine, Washington State University, 100 Grimes Way,
Pullman, WA 99164, USA; michael.court@wsu.edu

* Correspondence: mahajrod@gmail.com (S.K.); oleksyk@oakland.edu (T.K.O.); Tel.: +1-248-370-3359 (T.K.O)

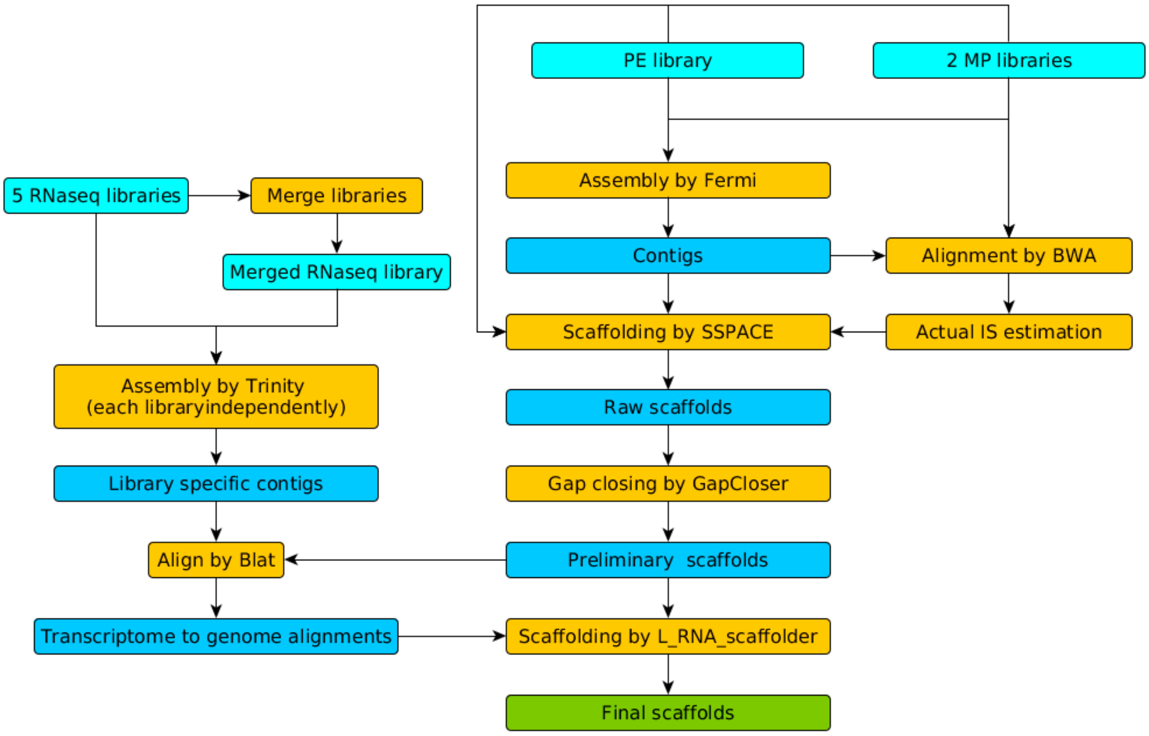

Figure S1. Pipeline used to assemble *A. vitatta* genome. One PE and two MP *A. vitatta* genome libraries were used to generate preliminary scaffolds complemented by additional scaffolding step using five *A. ventralis* RNAseq libraries.

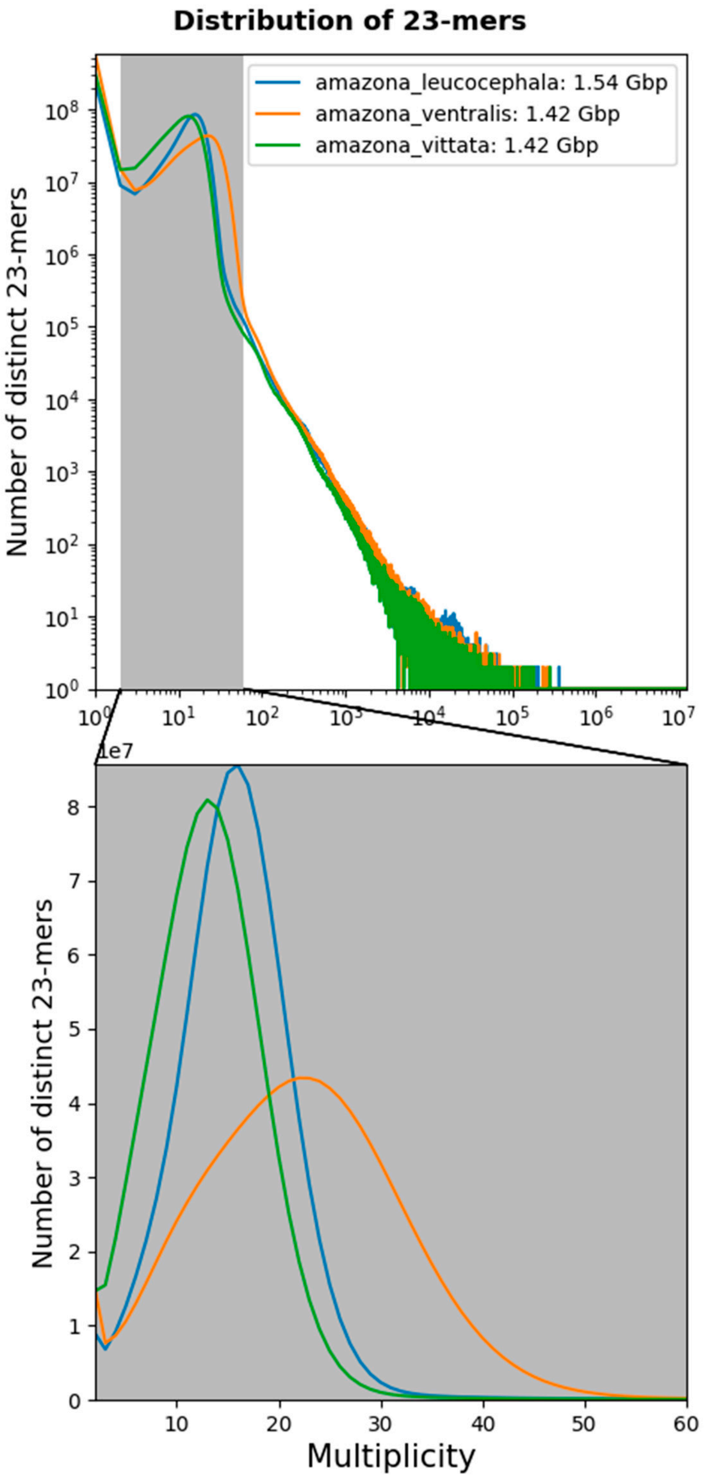

Figure S2. Distributions of 23-mers from PE libraries of three parrot species. Corresponding genome size estimation present in figure and also in Table 1. Only one major peak was detected for each genome, suggesting low heterozygosity level in all three species.

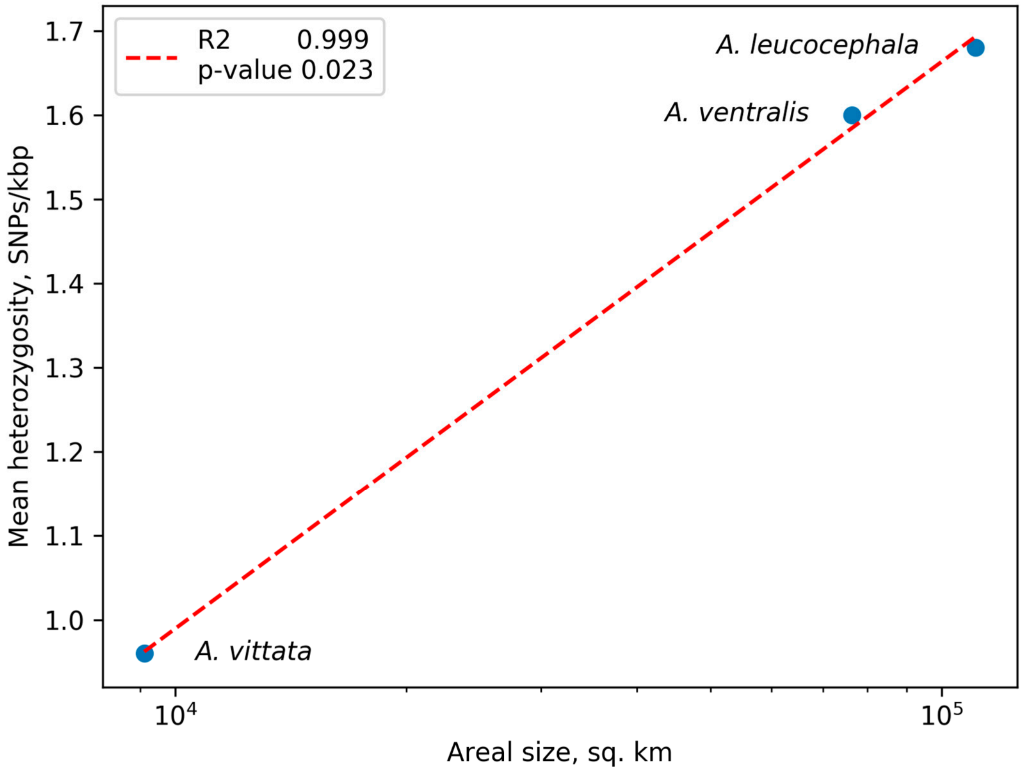

Figure S3. Connection between heterozygosity and areal size of the corresponding islands for the three *Amazona* species: *A. vittata* (Puerto Rico), *A. ventralis* (Hispaniola) and *A. leucocephala* (Cuba). The X axis is on the $\log_{10}$ scale, and a logarithmic trendline is displayed ($r^2 = 0.999$, p-value = 0.023)

Table S1. The sequencing outputs for each genome used in the current assemblies. Abbreviations: paired-end (PE) mate pairs (MP)

Species	Library Type	Actual Insert Size (bp)	Number of reads (M)	Total length (Gb)	Coverage (x)
A. vittata	PE	281bp	124.6	24.34	13
	MP	2,172	76.7	15.46	
	MP	6,935	292.5	38.81	
A. leucocephala	PE	281bp	159.9	31.94	16
A. ventralis	PE	281bp	204	40.82	22

Table S2. BUSCO scores for all steps of assembly of *A. vittata* genome. Assembly evaluation was performed using BUSCO v3 and *Avian* dataset (Simão et al. 2015).

	Complete	Complete and single-copy	Complete and duplicated	Fragmented	Missed
Fermi + SSPACE	82.6	82	0.6	8.4	9
Fermi + SSPACE + GapCloser	84.2	83.4	0.8	8.4	7.4
Fermi + SSPACE + GapCloser + L_RNA_scaffolder	87.4	86.5	0.9	6.4	6.2

Table S3. Available fossil-based calibrations for speciation time dating within *Psittaciformes* and Passeriformes clades. All ages are in millions of years (MYA)

**Split**	**Psittaciformes / Passeriformes**	***Manacus* / *Taeniopygia* / *Geospiza***	***Taeniopygia* / *Geospiza***
**Node min age**	53.5 MYA	13.6 MYA	7.2 MYA
**Node max age**	65 MYA	16.3 MYA	11.6 MYA
**Evidence**	fossil, biostratigraphy	fossil (distal humerus, proximal ulna, distal tarsometatarsus)	fossil (nearly complete postcranial skeleton)
**Reference**	(Ksepka and Clarke 2015)	(Jarvis et al. 2014)	(Jarvis et al. 2014)
**Oldest fossil taxon**	*Pulchrapollia gracilis*	*Miocitta galbreathi*	*Corvus larteri*
**Description**	Oldest known species of Psittaciformes, K-T boundary	Internal Branch "*Manacus*+*Corvus*+ +*Taeniopygia*+*Geospiza*" - MRCA of suboscines and oscines	Terminal Branch "*Corvus*" = MRCA "*Corvus*+(*Taeniopygia*,*Geospiza*)", Corvidae

Table S4. Mean heterozygosity in the genomes of three *Amazona* parrot species compared with that reported earlier for other species of birds .The table only contains species with fragmentation of genome assembly (N50) comparable to *A. vittata*.

Common name	Order	Conservation status	Whole Genome Heterozygosity
White-tailed Eagle	Accipitriformes	EV (Once VU)	0.00040
Dalmatian pelican	Pelecaniformes	EV (VU)	0.00060
Great black cormorant	Pelecaniformes	LC	0.00139
Turkey vulture	Accipitriformes	LC	0.00118
Kea	Psittaciformes	EV (VU)	0.00091
Puerto Rican Parrot	Psittaciformes §	EV	0.00096
Hispaniolan parrot	Psittaciformes §	NT	0.00160
Cuban parrot	Psittaciformes §	VU	0.00168

* [81]
§ this study
